# Mortality in HIV infected individuals in Pune, India

**Published:** 2011-04

**Authors:** Manisha Ghate, Swapna Deshpande, Srikanth Tripathy, Sheela Godbole, Madhura Nene, Madhuri Thakar, Arun Risbud, Robert Bollinger, Sanjay Mehendale

**Affiliations:** National AIDS Research Institute (ICMR), Pune, India; * *Johns Hopkins University,Baltimore, MD, USA*; ** *Present address*: National Institute of Epidemiology, Second Main Road, Tamil Nadu Housing Board, Ayapakkam, Chennai 600 077, India

**Keywords:** ART, CD4 count, HIV, immunosuppression, India, mortality

## Abstract

**Background & objectives:**

With the presence of HIV epidemic for more than two decades in India, rise in the number of HIV related deaths is expected. Data on mortality in HIV infected individuals from prospective studies are scanty in India. We report here data on mortality in a systematically followed cohort of HIV infected individuals at Pune, Maharashtra, India

**Methods:**

A total of 457 HIV infected individuals were enrolled in a prospective study in Pune between September 2002 and November 2004. They were evaluated clinically and monitored for CD4 counts at every quarterly visit. Mortality data were collected from the records of hospital facilities provided by the study. If the death occurred outside such hospitals; relatives of the participants were requested to inform about the death.

**Results:**

Median CD4 count in study participants was 218 cells/µl (95% CI: 107-373) at baseline. The median duration of follow up was 15 months (IQR: 12, 22). Mortality was higher in antiretroviral therapy (ART) naive patients compared to those who received treatment (16.59 vs. 7.25 per 100 person years). Participants above 35 yr of age, CD4 count less than or equal to 100 cells/µl at baseline, tuberculosis at any study time point and ART status were independently associated with high mortality [(RR=1.97; 95% CI: (1.23, 3.14), *P*=0.005, (RR=33.20, 95%CI (7.59, 145.29), *P*<0.001, (RR=2.38, 95% CI (1.38, 4.09), *P*= 0.002 and RR=5.60, 95% CI (3.18, 9.86), *P*<0.001, respectively].

**Interpretation & conclusions:**

High mortality at advanced immunosuppression highlights the importance of early detection of HIV infection. Emphasis needs to be given at timely diagnosis and management of tuberculosis and ART initiation. It is important to create awareness about availability of free antiretroviral drugs in the government ART roll out programme.

It has been estimated recently that approximately 2.47 million individuals are living with HIV infection in India^1^ and nearly 300,000 deaths due to AIDS have been reported by 2008^2^. Studies from developing countries have reported reduction in mortality in the post highly active antiretroviral therapy (HAART) era^3^,^4^. Government of India has initiated free antiretroviral therapy (ART) roll out programme since 2004. Currently, there are over 211 free ART centres all over the country providing antiretroviral therapy to 2, 20,000 HIV infected individuals (National AIDS Control Organization, unpublished data). Hence, it is expected that AIDS would be transformed from a fatal disease into chronic manageable condition in India. A study from south India has reported a dramatic decrease in mortality from 25 to 5 deaths per 100 person years in HIV infected individuals between 1997 and 2003 after initiation of HAART^5^. However, a retrospective study from Andhra Pradesh in southern India reported more deaths in HAART era as compared to pre HAART era^6^. This was attributed to less awareness of HIV status by patients and/or late diagnosis of HIV infection resulting in late initiation of ART. Thus, lack of access to ART continues to contribute to higher mortality in HIV infected individuals.

Though there are some reports on causes and prevalence of HIV related deaths in India^7^,^8^ very few reports are available on mortality on large cohorts followed for a longer period of time^9^,^10^. There could be problems in registration of deaths, more specifically, cause-specific mortality reporting. In many instances, the cause of death may be attributed to the opportunistic infections but there may not be a mention of HIV/AIDS status either due to failure to diagnose the underlying HIV disease or avoidance of mention of the underlying condition of HIV/AIDS due to confidentiality related concerns. An early report based on data from the death registry of the Mumbai Municipal Corporation mentioned that there was an increase in total number of deaths due to tuberculosis. It was assumed that a significant proportion of it could be AIDS-associated^11^.

Here we report the mortality in hospital and non-hospital settings in a systematically followed cohort of HIV infected individuals by demographic factors, ART status, opportunistic infections and the levels of immunosuppression represented by CD4 counts. We also report causes of death wherever available.

## Material and Methods

The study was approved by the Ethics Committee of the National AIDS Research Institute (NARI), Pune, India.

Under the umbrella of National Institutes of Health (NIH), USA sponsored HIV Prevention Trial Network (HPTN) study; National AIDS Research Institute conducted a prospective cohort study entitled ‘HIV incidence and participant retention’. The study was carried out in Pune city located in the high HIV prevalence State of Maharashtra in India. The study, initiated in September 2002, enrolled HIV serodiscordant couples (where one partner was HIV infected and other was not) with the objective of determining the incidence of HIV infection in the uninfected spouses at the end of one year. The study enrolment was continued until November 2004. Although originally designed as one year follow up study, the follow up was extended until August 2005 to accumulate more person years of follow up for determination of HIV incidence in uninfected partners in HIV serodiscordant couples.

*Patients and clinic based procedures*: HIV infected individuals were called for 3 monthly follow up visits. Demographic and clinical data were captured at each follow up visit. The clinical procedures in the study consisted of pre-test and post-test counselling, informed consent and collection of demographic and clinical information on structured proformas^12^. HIV infected participants were monitored for their CD4 counts and viral loads every three and six months respectively. Participants were advised to report to the study physicians for any illnesses whenever required. They were clinically evaluated by the study physicians during all follow up visits and during unscheduled interim visits. The study participants requiring in patient care were admitted at two pre-identified local hospitals and were jointly managed by the study and hospital physicians.

*Clinical management*: All patients were provided appropriate locally available standard clinical care for their illnesses. Trimethoprim-sulphamethoxazole prophylaxis was started once daily orally for preventing *Pneumocystis jirovecii* pneumonia (PJP) when CD4 counts fell below 200 cells/µl. Antiretroviral therapy was advised to patients as per guidelines provided by National AIDS Control Organisation (NACO)^13^ and was initiated in willing and affording patients after adherence counselling as free ART roll out programme was not started during the study period. The diagnoses of opportunistic infections were based on clinical presentations and/or supported by available laboratory investigations.

*Mortality data*: The mortality data were extracted from the hospital records and notes of the study clinicians were carefully reviewed. When deaths of patients were informed by their relatives; they were requested to show the death certificates to verify the cause of death and the study records were updated. Health outreach workers visited patients’ homes when the patients did not return for follow up visits and the information on mortality was collected. Clinical diagnoses at the time of death mentioned on the hospital and study records were considered for analysis.

*Statistical analysis*: Mortality per hundred person years with 95 per cent confidence intervals were computed for the cohort of 457 HIV infected patients by age, gender, presence of opportunistic infections, baseline CD4 count, viral load and ART status. Person years were calculated from the date of enrolment into the study to the date of last follow up or August 31, 2005; whichever was earlier for the censored cases, and date of death in case of patients who died.

To determine the risk factors associated with mortality, univariate and multivariate Poisson regression analyses were carried out. In the regression model, tuberculosis was considered as the primary opportunistic infection for analysis even though other co-morbidities were also present. Goodness of fit of model to the data was tested using ‘*estat gof*’ command in STATA. Various interaction terms were introduced and tested in Poisson models to test interaction effects on mortality. Means were compared using Student’s t-test if the data were approximately normally distributed; otherwise distributions were compared using nonparametric test. For comparison of proportions, the Chi square test was used. A two–tailed *P*<0.05 was considered statistically significant. Data were analyzed using STATA v10 (STATA corp., College Station, Texas, USA).

## Results

Data on 457 HIV infected individuals from serodiscordant couples were considered for analysis. The median duration of follow up was 15 months (IQR: 12, 22) and the median CD4 count was 218 cells/µl (IQR: 107-373). Male: female ratio in the enrolled study participants was 6:1. Of the total participants, 83 (18%) died during the study period with the mortality of 13.13 per 100 person years of follow up (95% CI: 10.46, 16.28). Mortality was higher in antiretroviral therapy (ART) naive patients compared to those who received treatment [16.59 per 100 person years of follow up (95% CI: 13.03, 21.11) vs. 7.25 (95% CI: 4.50, 11.65) per 100 person years].

The Table summarizes mortality by demographic, clinical and biological characteristics among the study participants. Mortality was higher in males [14.68 per 100 person years of follow up (95% CI: 11.79, 18.27)], participants in the age group of 35 yr and above [19.93 per 100 person years of follow up (95% CI: 15.27, 26.03)] and in those with presence of tuberculosis [26.62 per 100 years of follow up (95% CI: 19.74, 35.89)] or opportunistic infections other than TB [12.60 (95% CI: 7.94, 20.01)] at any study time point. Two hundred and twelve participants (46%) had CD4 count less than 200 cells/µl at the entry. The mortality was inversely proportional to the CD4 count and directly proportional to the viral load. It was highest in those with CD4 count of less than or equal to 100 cells/µl [42.06 per 100 person years (95% CI: 32.05, 55.20)] and viral load above 10,000 copies/ml [15.30 per 100 person years of follow up (95% CI: 12.23, 19.17)].

**Table.** Mortality per 100 PY and univariate and multivariate Poisson regression models to assess risk factors associated with mortality among HIV infected individuals in Pune, India

Characteristics	Deaths/n	PY†	Death / 100 PY*	Univariate models		Multivariate model	*P* value
				RR (95% CI)	*P* value	RR (95% CI)	
All	83 / 457	632	13.13 (10.58, 16.27)	-	-	-	-
Gender							
Female	3 / 64	87	3.43 (1.10, 10.63)	Reference		Reference	
Male	80 / 393	545	14.68 (11.79, 18.27)	4.28 (1.35, 13.56)	0.013	1.83 (0.57, 5.92)	0.311
Age (yr)							
< 35	29 / 257	362	8.01 (5.57, 11.54)	Reference		Reference	
≥ 35	54 / 200	271	19.93 (15.27, 26.03)	2.48 (1.58, 3.90)	0.001	1.97 (1.23, 3.14)	0.005
Opportunistic Infection							
Absent	22 / 243	328	6.70 (4.41, 10.18)	Reference		Reference	
Presence of TB	43 / 113	162	26.62 (19.74, 35.89)	3.97 (2.37, 6.64)	< 0.001	2.38 (1.38, 4.09)	0.002
Presence of OI other than TB	18 / 101	143	12.60 (7.94, 20.01)	1.88 (1.01, 3.51)	0.047	1.28 (0.68, 2.44)	0.445
Baseline CD4 count‡ (cells / µl)							
351+	2 / 128	189	1.06 ((0.26, 4.22)	Reference		Reference	
201-350	10 / 115	160	6.23 (3.35, 11.58)	5.90 (1.29, 26.93)	0.022	4.82 (1.03, 22.49)	0.046
101-200	18 / 102	155	11.61 (7.31, 18.43)	10.99 (2.55, 47.50)	0.001	7.72 (1.69, 35.19)	0.008
≤ 100	52 / 110	124	42.06 (32.05, 55.20)	39.84 (9.70, 160.53)	< 0.001	33.20 (7.59, 145.29)	< 0.001
Baseline viral load § (copies/ml)							
<10000	5 / 94	133	3.76 (1.56, 9.03)	Reference		Reference	
≥10000	76 / 359	496	15.30 (12.23, 19.17)	4.07 (1.64, 10.07)	0.002	1.34 (0.52, 3.47)	0.553
ART status							
ART received	17 / 141	235	7.25 (4.50, 11.65)	Reference		Reference	
ART naive	66 / 398	398	16.59 (13.03, 21.11)	2.29 (1.34, 3.90)	0.002	5.60 (3.18, 9.86)	< 0.001

* 95 % CI denotes confidence interval and calculated using ‘Byar approximation to Poisson’; PY, person years; OI, opportunistic infections; ART, anti retroviral therapy; TB, tuberculosis (pulmonary and/or extra pulmonary);

† Person years were computed to two decimal places and were rounded to whole numbers;

‡ Baseline CD4 count available in 455 patients,

§ baseline viral load available in 453 patients

Multivariate analysis of mortality among HIV infected individuals showed that participants above 35 yr of age, CD4 count less than or equal to 100 cells/µl at baseline, presence of tuberculosis at any study time point and ART naive were independently associated with high mortality [(RR=1.97; 95% CI: (1.23, 3.14), *P*=0.005, (RR=33.20, 95%CI (7.59, 145.29), *P*<0.001, (RR=2.38, 95% CI (1.38, 4.09), *P*=0.002 and RR=5.60, 95% CI (3.18, 9.86), *P*<0.001, respectively]. Gender, opportunistic infection other than tuberculosis and viral load were not related to mortality in multivariate analysis (Table). Goodness of model fit was tested using model’s Chi square value and corresponding *P* value (chi square = 265.45, *P*=0.999). Important interaction terms such as age*CD4 count (*P*=0.98), viral Load*CD4 count (*P*~0.99 for all levels of interaction), viral load*age (*P*=0.93) and ART status* opportunistic infection status (*P*~0.90 for all levels of interaction) were also included. All the interactions were non-significant and were not included into the final model of multivariate analysis. Mortality considering last CD4 count as a covariate was also included. Last CD4 was available for 439 of 457 HIV infected participants. The mortality was inversely associated with the last CD4 count. It was highest among those who had CD4 count less than 100 cells/µl [RR=23.6, (95% CI: 5.75, 96.51), *P*<0.001] (data not shown).

Causes of death were ascertainable in 49 (59%) out of 83 total deaths; but it could not be obtained in case of the remaining patients as their relatives could not provide details or show death certificates to confirm the cause of deaths. Of these 49, 24 (49%) patients had pulmonary or extra-pulmonary tuberculosis prior to death and their baseline median CD4 count was 67 cells/µl (IQR: 42-107). Twenty three (47%) patients had TB unrelated causes (toxoplasmosis, cryptococcal meningitis, septicaemia, *Pneumocystis jirovecii* pneumonia, acute gastroenteritis, disseminated intravascular coagulation, myocardial infarction, lactic acidosis, progressive multifocal leucoencephalopathy, and hepatorenal syndrome) and their baseline median CD4 count was 96 cells/µl (IQR: 50-231). The above causes were mentioned on source documents available prior to death or death certificates either from pre-identified hospitals or from relatives of the patients. Two patients had HIV unrelated mortality.

The causes of death could not be obtained in 34 patients. The baseline median CD4 count in the cases with unknown cause was [76 cells/µl (IQR: 59-117)]. The person time of follow up was also less in these patients (27 PY) as compared to those with known causes of death (39 PY).

Data on mortality among patients who were admitted in the hospital for inpatient care were analyzed to know their treatment seeking behaviour at the time of death. Out of 83 patients, only 28 (33%) were admitted in the pre - determined hospitals prior to death.

## Discussion

The data presented here provide information on mortality among 457 HIV infected individuals in Pune. They participated in an observational study and had free access to non ART treatment. Antiretroviral drugs were advised as per guidelines provided by National AIDS Control Organisation (NACO)^13^ but could not be provided free of cost as they were not a part of standard of treatment care at that time point. HIV associated mortality has been described previously through natural history^5^,^10^ and hospitalized patients’ studies^6^–^8^ from India. The present study has an added advantage of reporting the mortality in a systematically followed cohort of persons with HIV infection and provides the data by various socio-demographic, ART status and disease progression related factors. It was observed that higher age, lower CD4 counts at baseline, presence of tuberculosis at any study time point and ART naive status were significantly associated with mortality.

Mortality was significantly higher in older age group (> 35 yr). This finding suggests that age itself is a poor prognostic factor. It was significantly related to low CD4 counts which highlights bad prognosis in patients with advanced stage of HIV disease. A study from Brazil has shown that patients with AIDS at entry were 5 times more likely to die as compared to patients who were asymptomatic at entry^14^. Data from Jamaica have shown similar findings that age, number of opportunistic diseases, and initial stage were strongly associated with mortality^15^. Our findings showed that mortality was significantly higher in ART naive patients as compared to those who received ART. The mortality reported in ART naive participants was less than that reported from a study in southern India in pre HAART era (25 per 100 person years)^5^. This may be due to the difference in the profile of study population at entry, study design and differential follow up rates. The overall mortality in our cohort was lower than that observed in the hospitalized patients from Pune city (26.3%)^8^. However, the later study was carried out in a tertiary care government hospital wherein a large number of patients could have been admitted at terminal stages due to various illnesses attributing to higher mortality. Mortality in our study was lower than those reported from Sub-Saharan Africa (8 – 26%)^16^ and higher than that from Taiwan in pre HAART era^3^ (10.2 per 100 person years). A collaboration of 12 prospective cohort studies from Europe and the United States has shown that combined ART halved the average mortality rate in HIV-infected individuals^17^. The study from Sub-Saharan Africa has reported early mortality among adults accessing antiretroviral treatment programmes primarily because of late initiation of ART in this population due to tuberculosis, acute sepsis, cryptococcal meningitis, malignancy and wasting syndrome as leading causes^16^. This stresses the importance of creating awareness among HIV infected persons and their health care providers about early detection of the opportunistic infections and timely treatment which can reduce or prevent morbidity and mortality before advanced immunosuppression occurs.

Our finding that tuberculosis was the commonest opportunistic infection seen in patients who died was similar to that reported from other parts of India^7^–^9^. Considering the high rates of HIV-TB co-infection and morbidity and mortality associated with it, early detection and management of HIV as well as tuberculosis gain importance as significant public health interventions in the National AIDS Control Program (NACP). Recent revision in the National guidelines recommends initiation of antitubercular treatment (ATT) in HIV-TB co-infected patients with CD4 count of 350 cells/µl. It has also been mentioned that antiretroviral drugs could be initiated in these patients after two weeks of starting ATT^18^. A study from Brazil has reported dramatic decrease in incidence of tuberculosis when timely HIV diagnosis and treatment were introduced^19^. It has been reported that early HAART (within 2 months of TB treatment) is favoured as compared to deferred and late HAART to reduce the mortality^20^. Systematic studies should be planned to observe the effect of timing of HAART initiation in our set up and to study the mortality outcomes in HIV-TB co-infected patients. Studies are currently ongoing in India that could provide guidance about prophylaxis to prevent tuberculosis among HIV infected individuals. If proved to be successful, anti-TB chemoprophylaxis could significantly reduce morbidity and mortality associated with TB in HIV infection.

The mortality analysis presented here has certain limitations. The number of women in this cohort was small so elaborate gender specific analysis could not be performed. The follow up was short term. Though 39 patients had history of receiving ART at baseline and 102 were initiated with ART at some time point during study period, we were unable to capture the details of ART drug regimens and adherence among them which could influence the study end points. Possibility of non compliance in these patients cannot be ruled out. The mortality rates could not be estimated before and after ART initiation as exact date of ART initiation was not available in this observational study. The diagnoses of opportunistic infections were based on clinical examination supported by laboratory investigations or response to therapy in some conditions. However, these results reflect a ‘real life situation’ in a developing country. Nearly half of the patients did not have cause of death data; and in those who had, it was not clear how precise those data were.

Cause of death could not be ascertained in about 41 per cent of the total deaths. It may be due to the fact that there may be significant movement of HIV infected individuals to their villages with advanced stage of disease. This may have reduced the access to good health care and resulted in deaths where causes could not have been ascertained. If these individuals would have remained under medical care provided by the study, some of the deaths could have been prevented. We could not collect systematic data on reasons for not reporting the terminal illnesses or deaths to our clinic but some of the common reasons verbally reported to the study staff by the relatives were, less manpower to attend to the patient in hospital due to other family responsibilities and moving away from the local residence to native place in the rural area to avoid stigma associated with the disease. This issue has been addressed in the National AIDS Control Programme - III (NACP - III) in which adequate stress has been given on outreach activity, monitoring and tracking of HIV infected individuals. The concept of Community Care Centres (CCC) under NACP - III has envisaged that these centres would provide comprehensive support to HIV infected individuals. It is expected that CCC would play a critical role in enabling them to access ART as well as providing monitoring, follow up and counselling support. This would help in reporting morbidity and mortality in HIV infected individuals^21^.

In conclusion, this study provides important information about mortality in HIV infected individuals. It highlights the importance of creating awareness about early diagnosis of HIV infection among health care providers. Educating health care providers and people about importance of early treatment and referral to ART centres would go a long way in reducing the mortality in HIV infected patients.
